# Antrum Preservation Versus Antrum Resection in Laparoscopic Sleeve Gastrectomy With Effects on Gastric Emptying, Body Mass Index, and Type II Diabetes Remission in Diabetic Patients With Body Mass Index 30–40 kg/m^2^: a Randomized Controlled Study

**DOI:** 10.1007/s11695-022-05982-5

**Published:** 2022-03-19

**Authors:** Moheb S. Eskandaros

**Affiliations:** Department of General Surgery, Faculty of Medicine, Ain Shams University, Cairo, Egypt

**Keywords:** Sleeve gastrectomy, Antral resection, Antral preservation, Gastric emptying, BMI

## Abstract

**Background:**

Laparoscopic sleeve gastrectomy (LSG) is a widely performed procedure nowadays. There is a controversy on whether antrum resection (AR) or antrum preservation (AP) should be done and if this has an effect on BMI, gastric emptying, and associated medical conditions such as diabetes mellitus (DM).

**Study Design:**

This randomized controlled trial included 56 patients in the AP group and 53 patients in the AR group with BMI 30–40 kg/m^2^. Weight, BMI, fasting and postprandial blood glucose (FBS and PPBS), HbA1C, oral hypoglycemic drug use, and % gastric emptying by gastric scintigraphy at 30, 60, 90, and 120 min were recorded preoperatively and postoperatively at 3, 6, and 12 months. Postoperative % of total weight loss (TWL) and symptoms of de novo GERD were observed at 3, 6, and 12 months.

**Results:**

The AR group had significantly lower BMI and HbA1C and higher %TWL than the AP group. There was a significant difference between the two groups regarding % of gastric emptying with the AP group showing higher values at 30, 60, 90, and 120 min. There were no significant differences regarding FBS, PPBS, and oral hypoglycemic use. The AR group had more incidence of GERD symptoms postoperatively yet with no significant difference.

**Conclusion:**

LSG with antrum resection (2 cm from the pylorus) had significantly less postoperative BMI, higher %TWL, better control of type II DM, and more retention of gastric contents in patients with BMI 30–40 kg/m^2^ in comparison with LSG with antral preservation with non-significant increase in incidence of GERD symptoms.

**Graphical abstract:**

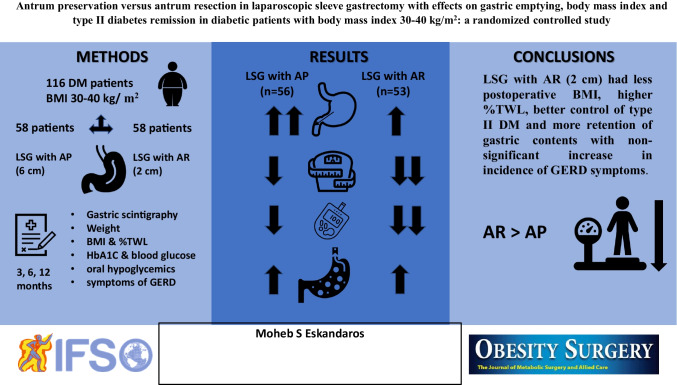

## Introduction

The laparoscopic sleeve gastrectomy (LSG) is a very popular bariatric procedure worldwide. It has been recommended by many bariatric surgeons as a stand-alone procedure due to its feasibility and good results in terms of weight loss and in remission of metabolic conditions such as type II diabetes mellitus (DM) [1, 2]. The main mechanism for weight loss is the restrictive nature of the procedure in addition to its effect on the gastrointestinal hormones [3, 4] and on the gastric emptying [5]. LSG has been proved to help in controlling DM and drive the type II diabetic patients into remission.

Many studies were conducted on the effect of LSG on the gastric emptying with some studies showing decreased gastric emptying leading to the early sense of satiety that shares in the mechanisms of weight loss after LSG while others reported increase in the gastric emptying [6–9] leaving the restrictive mechanism as the sole effect in decreasing weight. Some surgeons advocate to leave the antrum intact and resect the stomach above it while others resort to resecting the antrum along with the rest of the stomach. This leads to controversy if resecting the antrum has an effect on the gastric emptying [10–13] and whether resecting the antrum helps in preventing weight regain and maintaining the remission of associated medical conditions such as DM [14, 15]. Also, to our knowledge, no studies were performed on the difference of the effect of antrum preservation versus antrum resection on gastric emptying in type II diabetes mellitus patients.

In this study, we tried to detect by evidence the effect of antral preservation (AP) (by starting resection at 6 cm from the pylorus using 36 F bougie) versus antral resection (AR) (by starting resection at 2 cm from the pylorus using 36 F bougie) on the gastric emptying after LSG in diabetic patients of body mass index (BMI) 30–40 kg/m^2^ and following them up at 3, 6, and 12 months.

## Patients and Methods

This is a randomized controlled trial (RCT) that took place from May 2017 till May 2020 in the Department of General and Bariatric Surgery, Faculty of Medicine, Ain Shams University, Cairo, Egypt. The patients were followed up for 12 months till May 2021. The patients were recruited from the bariatric outpatient clinic.

The inclusion criteria were diabetic patients of BMI 30–40 kg/m^2^ and undergoing sleeve gastrectomy. The exclusion criteria were previous bariatric or upper gastrointestinal surgeries, patients with gastroesophageal reflux disease (GERD) by upper endoscopy preoperatively, patients on medications that affect gastric motility, DiaRem score > 12 (only patients on oral hypoglycemics were included to avoid preoperative DM severity interfering with the results) or unfit for surgery.

### Sample Size Calculation

In the study by Vives et al. [15], the authors compared antral resection (3 cm from the pylorus) and antral preservation (8 cm from the pylorus) regarding gastric emptying, BMI, and remission of metabolic syndrome in diabetic and non-diabetic patients (total number of patients were 60 patients divided into two groups). Assuming the endpoint of T 1/2 (time taken for the stomach to empty half of its contents) at 12 months in diabetic patients with antral resection which was 39 ± 7.6 min in their study and assuming the average T 1/2 in our study would be 44 min (5 min difference or 12.5% difference) at 12 months in diabetic patients with antral resection 2 cm from the pylorus and having a power of at least 90% and alpha error 0.05, the minimum number of patients will be 98 patients.

### Patient Enrollment

The overall patients who were candidates for LSG in the study period were 1396 patients. The type II diabetic patients with BMI 30–40 kg/m^2^ and fulfilling the inclusion criteria were 459 patients. DiaRem score was done and patients with score ≤ 12 were enrolled in the study with equal chance for remission of DM for all patients guided by previous studies [15, 16] and these were found to be 116 patients. These were divided into 2 groups: antrum preserving group (AP) as a control group and antrum resection group (AR) as the study group with each including 58 patients.

### Randomization

Allocation of patients in either group was performed by computer program for randomization.

Each patient underwent routine preoperative investigations in addition to preoperative gastric scintigraphy to detect the time for gastric emptying.

### Gastric Emptying Scintigraphy

The patients were instructed to be fasting overnight (9 h at least) with cessation of proton pump inhibitors 3 days before study along with any other medication that affects the gastric emptying if present. All patients were asked to consume a standard semi-solid meal of 150 ml labeled with Tc-99 m. Patients were kept in semi-sitting position. A gamma camera was used to detect the percent of gastric emptying at 30, 60, 90, and 120 min.

### Surgical Procedure

All procedures were performed laparoscopically using 5-port technique. The gastric pouch was fashioned using a 36 F bougie. The greater omentum was dissected from the greater curvature using Ligasure™ (Covidien, Dublin, Ireland). The pouch was created by applying Endo GIA Tri-Stapler (Covidien, Dublin, Ireland) using 60 mm cartridges. In the antrum preserving group (AP), stapling started at 6 cm proximal to the pylorus while in the antrum resection group (AR), stapling started at 2 cm proximal to the pylorus. Stapling was continued guided by the bougie applied to the lesser curvature and ending at 1 cm to the left of the angle of His. Hemostasis was done. Test for staple line integrity by methylene blue test was performed. An 18 F drain was left in the surgical bed.

All patients received anticoagulation 24 h after surgery with early ambulation. All patients underwent gastrografin contrast meal before starting oral intake to detect any signs of leak. Patients with GERD symptoms postoperatively were instructed to use PPI (proton pump inhibitors).

### Data Collection

Preoperative weight, height, BMI, fasting and postprandial blood glucose, HbA1C, and oral hypoglycemic drug use were recorded. Preoperative percent of gastric emptying by gastric scintigraphy at 30, 60, 90, and 120 min was recorded. Operative time and complications were detected.

Postoperative gastric scintigraphy results at 30, 60, 90, and 120 min (primary outcome), weight, BMI, % of total weight loss (TWL), fasting and postprandial blood glucose, HbA1C, oral hypoglycemic status, and symptoms of de novo GERD (secondary outcomes) (assessment was carried out by using patient assessment of upper gastrointestinal disorder-symptom severity index (PAGI-SYM) standardized questionnaire scored on a 6-point Likert scale, with response options ranging from 0 (none) to 5 (very severe)) were observed at 3, 6, and 12 months.

### Data Analysis

The collected data were revised, coded, tabulated, and introduced to a PC using IBM SPSS Statistics for Windows, version 26 (IBM Corp., Armonk, NY, USA). Data was presented and suitable analysis was done according to the type of data obtained for each parameter.

#### Descriptive Statistics

1. Mean, standard deviation (± SD), and range for parametric numerical data, while median and interquartile range (IQR) for non-parametric numerical data.

2. Frequency and percentage of non-numerical data.

#### Analytical Statistics

1. Independent *t* test was used to assess statistical significance of the difference between two group means.
2. Pearson’s chi-square test was used to examine the relationship between two qualitative variables.
3. Fisher’s exact test was used to examine the relationship between two qualitative variables when the expected count is less than 5 in more than 20% of cells.
4. Repeated measure ANOVA (Greenhouse–Geisser correction was applied if Mauchly’s assumption of sphericity was violated) was used to assess the statistical significance of the difference between means measured more than twice within study groups.
5. Partial eta squared (ηρ^2^) was used to detect effect size within groups.
6. Post hoc test was used to detect the statistical significance between each mean pair within the same group.
7. Linear regression with linear fit was used to detect values of *y*-axis within certain point of time on *x*-axis using linear fit of the curve.
8. Logistic regression was used to predict the probability of association of one dichotomous variable with another continuous or categorical variable.

A *p* value < 0.05 was considered significant.

## Results

This randomized controlled trial (RCT) included 58 patients in each group with AP group considered control group and AR group considered test group. The follow-up was done at 3, 6, and 12 months postoperatively. Two patients in the AP group and 5 patients in the AR group did not complete the follow-up period (dropout) after 3 months so they were excluded. The remaining 56 patients in the AP group and 53 patients in the AR group yielded the following results:

### Demographic and preoperative data

There were no significant differences between the two groups regarding the age, height, weight, BMI, fasting blood glucose (FBS), postprandial blood glucose (PPBS), glycated hemoglobin (HbA1C), percent of preoperative gastric emptying scintigraphy at 30, 60, 90, and 120 min. There were no significant differences between the two groups regarding the sex distribution and oral hypoglycemic drug use (either metformin or sulfonyl urea derivatives) with no insulin among patients (all patients have DiaRem score ≤ 12 and insulin use alone scores 10 points). The operative time had no significant differences between the two groups. No major complications were recorded in the immediate postoperative period. These data are summarized in Table 1.

**Table 1:** Demographic data and preoperative age, height, weight, BMI, FBS, PPBS, HbA1C, % of preoperative gastric emptying scintigraphy at 30, 60, 90, and 120 min, sex distribution, and oral hypoglycemic drug use

		AP (*n* = 56)		AR (*n* = 53)		*p* value^*^
		Mean	SD	Mean	SD	
Age		45.11	8.34	46.08	9.11	0.565
Height		1.69	0.06	1.69	0.06	0.510
Weight		110.80	9.30	110.79	8.18	0.995
BMI		38.70	0.94	38.35	1.61	0.182
FBS		117.96	12.43	119.42	12.84	0.551
PPBS		177.61	18.88	179.75	15.75	0.520
HbA1C		7.06	0.47	6.97	0.43	0.303
Preop. % gastric emptying (0.5 h)		28.16	7.72	28.20	6.94	0.973
Preop. % gastric emptying (1 h)		52.42	2.57	52.33	2.44	0.854
Preop. % gastric emptying (1.5 h)		67.35	3.22	67.03	3.33	0.612
Preop. % gastric emptying (2 h)		86.89	4.70	86.83	4.75	0.945
Operative time		65.79	8.45	65.06	9.39	0.672
		*N*	%	*N*	%	
Sex	Female	43	76.8%	39	73.6%	0.699
Oral hypoglycemics	Metformin	47	83.9%	45	84.9%	0.888
	Sulfonylurea	9	16.1%	8	15.1%	

^*^t (independent *t* test) and *χ*^2^ (Pearson’s chi-square test). *p* value < 0.05 was considered statistically significant

### Gastric Emptying

There were significant differences (*p* value 0.001 or less) between the two groups regarding % of gastric emptying at 3 months with the AP group showing higher values at 30, 60, 90, and 120 min in comparison to the AR group (37 ± 1.97%, 63.58 ± 3.07%, 73 ± 3.16%, and 91.41 ± 4.25% in the AP group versus 34 ± 2.11%, 60.71 ± 2.76%, 70.88 ± 3.14%, and 88.28 ± 5.16% in the AR group respectively) as shown in Table 2.

**Table 2:** Three-month follow-up on weight, BMI, %TWL, FBS, PPBS, HbA1C, % of gastric emptying scintigraphy at 30, 60, 90, and 120 min, GERD symptoms, and oral hypoglycemic drug use

3 months		AP (*n* = 56)		AR (*n* = 53)		*p* value^*^
		Mean	SD	Mean	SD	
Weight		100.52	9.52	97.30	8.45	0.065
BMI		35.08	1.13	33.66	1.68	0.000
%TWL		9.35	1.54	12.24	1.88	0.000
FBS		102.36	12.55	98.23	13.79	0.106
PPBS		143.45	19.07	138.87	16.12	0.178
HbA1C		6.40	0.49	6.17	0.44	0.013
3 months % gastric emptying (0.5 h)		37.00	1.97	34.00	2.11	0.000
3 months % gastric emptying (1 h)		63.58	3.07	60.71	2.76	0.000
3 months % gastric emptying (1.5 h)		73.00	3.16	70.88	3.14	0.001
3 months % gastric emptying (2 h)		91.41	4.25	88.28	5.16	0.001
		*N*	%	*N*	%	
GERD symptoms	Yes	6	10.71%	10	18.87%	0.229
	No	50	89.29%	43	81.13%	
Oral hypoglycemics	Metformin	27	48.21%	29	54.71%	0.861
	Sulfonylurea	5	8.92%	4	7.54%	
	No	24	42.87%	20	37.75%	

^*^t (independent *t* test) and *χ*^2^ (Pearson’s chi-square test). *p* value < 0.05 was considered statistically significant

At 6 months, there were significant differences (*p* value < 0.001) between the two groups regarding % of gastric emptying with the AP group showing higher values at 30, 60, 90, and 120 min in comparison to the AR group (38.55 ± 1.99%, 65.64 ± 2.75%, 75.03 ± 3.44%, and 91.76 ± 4.56% in the AP group versus 35.01 ± 1.99%, 61.75 ± 2.54%, 70.69 ± 2.99%, and 88.13 ± 3.85% in the AR group respectively) as shown in Table 3.

**Table 3:** Six-month follow-up on weight, BMI, %TWL, FBS, PPBS, HbA1C, % of gastric emptying scintigraphy at 30, 60, 90, and 120 min, GERD symptoms, and oral hypoglycemic drug use

6 months		AP (*n* = 56)		AR (*n* = 53)		*p* value^*^
		Mean	SD	Mean	SD	
Weight		91.77	9.55	87.36	8.70	0.013
BMI		32.01	1.27	30.20	1.88	0.000
%TWL		17.29	2.34	21.27	2.76	0.000
FBS		93.91	12.02	90.53	12.48	0.153
PPBS		126.64	19.09	123.19	15.37	0.299
HbA1C		6.20	0.49	5.87	0.45	0.000
6 months % gastric emptying (0.5 h)		38.55	1.99	35.01	1.99	0.000
6 months % gastric emptying (1 h)		65.64	2.75	61.75	2.54	0.000
6 months % gastric emptying (1.5 h)		75.03	3.44	70.69	2.99	0.000
6 months % gastric emptying (2 h)		91.76	4.56	88.13	3.85	0.000
		*N*	%	*N*	%	
GERD symptoms	Yes	2	3.57%	6	11.32%	0.118
	No	54	96.43%	47	88.68%	
Oral hypoglycemics	Metformin	22	39.28%	21	39.62%	0.514
	Sulfonylurea	4	7.14%	1	1.88%	
	No	30	53.58%	31	58.5%	

^*^t (independent *t* test) and Fisher exact test. *p* value < 0.05 was considered statistically significant

At 12 months, there were significant differences (*p* value < 0.001) between the two groups regarding % of gastric emptying with AP group showing higher values at 30, 60, 90, and 120 min in comparison to the AR group (39.78 ± 1.99%, 66.87 ± 2.47%, 76.48 ± 3.18%, and 92.62 ± 3.85% in the AP group versus 36.18 ± 1.94%, 62.81 ± 2.97%, 71.73 ± 3.19%, and 89.73 ± 3.84% in the AR group respectively) as shown in Table 4.

**Table 4:** Twelve-month follow-up on weight, BMI, %TWL, FBS, PPBS, HbA1C, % of gastric emptying scintigraphy at 30, 60, 90, and 120 min, GERD symptoms, and oral hypoglycemic drug use

12 months		AP (*n* = 56)		AR (*n* = 53)		*p* value^*^
		Mean	SD	Mean	SD	
Weight		79.32	9.62	72.53	9.24	0.000
BMI		27.63	1.55	25.04	2.09	0.000
%TWL		28.60	3.30	34.76	4.12	0.000
FBS		86.82	12.99	83.55	12.83	0.189
PPBS		123.14	19.07	121.53	15.89	0.631
HbA1C		5.86	0.46	5.42	0.44	0.000
12 months % gastric emptying (0.5 h)		39.78	1.99	36.18	1.94	0.000
12 months % gastric emptying (1 h)		66.87	2.47	62.81	2.97	0.000
12 months % gastric emptying (1.5 h)		76.48	3.18	71.73	3.19	0.000
12 months % gastric emptying (2 h)		92.62	3.85	89.73	3.84	0.000
		*N*	%	*N*	%	
GERD symptoms	Yes	1	1.78%	3	5.66%	0.354
	No	55	98.22%	50	94.34%	
Oral hypoglycemics	Metformin	10	17.85%	11	20.75%	0.809
	Sulfonylurea	0	0%	0	0%	
	No	46	82.15%	42	79.25%	

^*^t (independent t test), Fisher exact test, and *χ*^2^ (Pearson’s chi-square test). *p* value < 0.05 was considered statistically significant

**Fig. 1:**
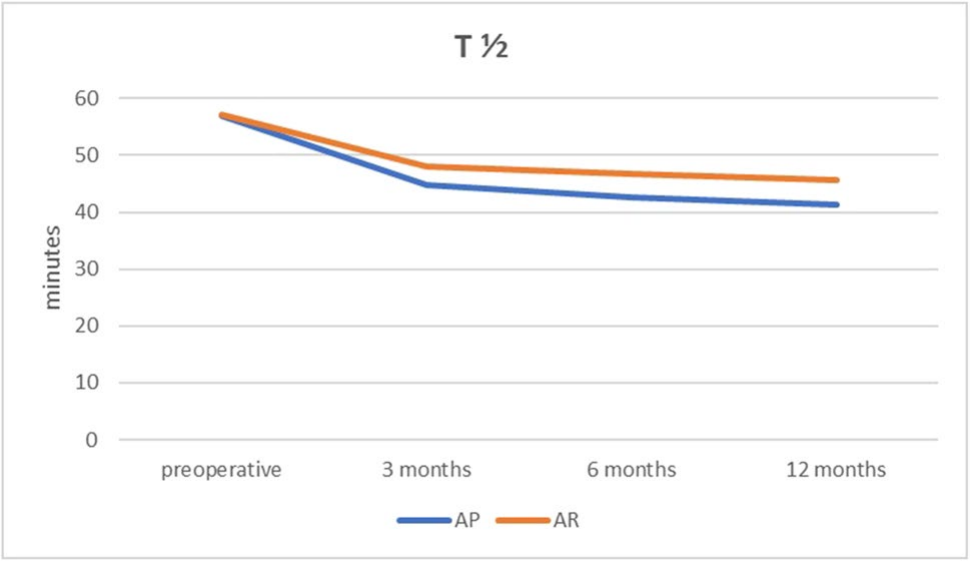
Graphic representation of T 1/2 (time required in minutes till 50% of ingested standard meal had been emptied) preoperatively and at 3, 6, and 12 months postoperatively

In order to facilitate comparison between the 2 groups, T 1/2 (elapsed time from consumption of the meal till 50% of the meal was emptied by the stomach) was calculated using linear regression with linear fit of the curve. Both groups showed faster evacuation postoperatively in comparison to the preoperative values. The AP group required less time than the AR group to empty 50% of the meal consumed at 3, 6, and 12 months as shown in Fig. 1. *p* value was 0.02 by repeated measure ANOVA which was considered statistically significant (< 0.05).

### BMI, %TWL, Metabolic Parameters, and GERD

At 3-month follow-up, there was no significant difference in weight (*p* value 0.065) but there was a significant difference regarding BMI and %TWL (*p* value < 0.001) with the AR group showing lower BMI (33.66 ± 1.68 kg/m^2^) and higher %TWL (12.24 ± 1.88%) in comparison with the AP group (35.08 ± 1.13 kg/m^2^ and 9.35 ± 1.54% respectively) as shown in Table 2. At 6-month follow-up, there was a significant difference in weight (*p* value 0.013) with the AR group having lower weight in comparison with the AP group (87.36 ± 8.70 kg versus 91.77 ± 9.55 kg respectively). There was a significant difference regarding BMI and %TWL (*p* value < 0.001) with the AR group showing lower BMI (30.20 ± 1.88 kg/m^2^) and higher %TWL (21.27 ± 2.76%) in comparison with the AP group (32.01 ± 1.27 kg/m^2^ and 17.29 ± 2.34% respectively) as shown in Table 3. At 12-month follow-up, there was a significant difference in weight (*p* value < 0.001) with the AR group having lower weight in comparison with the AP group (72.53 ± 9.24 kg versus 79.32 ± 9.62 kg respectively). There was a significant difference regarding BMI and %TWL (*p* value < 0.001) with the AR group showing lower BMI (25.04 ± 2.09 kg/m^2^) and higher %TWL (34.76 ± 4.12%) in comparison with the AP group (27.63 ± 1.55 kg/m^2^ and 28.60 ± 3.30% respectively) as shown in Table 4.

At 3 months, there were no significant differences between the two groups regarding FBS and PPBS with *p* values 0.106 and 0.178 respectively. There was significant difference regarding HbA1C (*p* value 0.013) with the AR group showing lower HbA1C (6.17 ± 0.44 gm%) in comparison with the AP group (6.4 ± 0.49 gm%) as shown in Table 2. There were no significant differences between the two groups regarding FBS and PPBS at 6 months with *p* values 0.153 and 0.299 respectively. There was a significant difference regarding HbA1C (*p* value < 0.001) with the AR group showing lower HbA1C (5.87 ± 0.45 gm%) in comparison with the AP group (6.2 ± 0.49 gm%) as shown in Table 3. There were no significant differences between the two groups regarding FBS and PPBS at 12 months with *p* values 0.189 and 0.631 respectively. There was a significant difference regarding HbA1C (*p* value < 0.001) with the AR group showing lower HbA1C (5.42 ± 0.44 gm%) in comparison with the AP group (5.86 ± 0.46 gm%) as shown in Table 4.

At 3 months, the AR group had more incidence of GERD symptoms postoperatively in comparison with the AP group (18.87% versus 10.71%) yet with no significant difference (*p* value 0.229). Both groups had decrease in the percent of patients taking oral hypoglycemics (57.13% in AP and 62.25% in AR) but with no significant difference (*p* value 0.861) as shown in Table 2. At 6 months, the AR group had more incidence of GERD symptoms in comparison with the AP group (11.32% versus 3.57%) yet with no significant difference (*p* value 0.118). Both groups had decrease in the percent of patients taking oral hypoglycemics (46.42% in AP and 41.5% in AR) but with no significant difference (*p* value 0.514) as shown in Table 3. At 12 months, the AR group had more incidence of GERD symptoms in comparison with the AP group (5.66% versus 1.78%) yet with no significant difference (*p* value 0.354). Both groups had decrease in the percent of patients taking oral hypoglycemics (only metformin) (17.85% in AP and 20.75% in AR) but with no significant difference (*p* value 0.809) as shown in Table 4.

There was a statistically high significant effect (*p* < 0.001) of time by repeated measure ANOVA (Greenhouse–Geisser correction was applied if Mauchly’s assumption of sphericity was violated) on weight, BMI, %TWL, FBS, PPBS, HbA1C, and % of gastric emptying at 30, 60, 90, and 120 min within the AP group and a statistically high significant effect (*p* < 0.001) of time on weight, BMI, %TWL, FBS, PPBS, HbA1C, and % of gastric emptying at 30, 60, and 90 and statistically significant effect (*p* < 0.05) on % of gastric emptying at 120 min within the AR group.

Estimation of effect size by partial eta squared (ηρ^2^) showed a large effect within subjects (both AP and AR groups) and the observed power was 1 in all variables except for % of gastric emptying at 120 min in the AR group which was 0.812.

Post hoc test with Bonferroni correction was applied for each variable at each two successive points of time rendering significant differences (*p* < 0.001) from a time point to the next in weight (all pairs of time points in both groups), BMI (all pairs of time points in both groups), %TWL (all pairs of time points in both groups), FBS (all pairs of time points in both groups), PPBS (all pairs of time points in both groups except 6–12 m in AR *p* 0.004), HbA1C (all pairs of time points in both groups), and % of gastric emptying at 30, 60, 90, and 120 min (at 0–3 m in both groups except in 120 min at 0–3 in the AR group). Post hoc test rendered significant differences in % of gastric emptying at 30 (*p* 0.001 at 3–6 m and 0.014 at 6–12 m in the AP group and 0.005 at 6–12 m in the AR group), 60 (*p* 0.004 at 3–6 m in the AP group), and 90 (*p* 0.012 at 3–6 m in the AP group). Non-significant results were obtained by post hoc test in % of gastric emptying at 30 (at 3–6 m in the AR group), 60 (at 3–6 m in the AR group and at 6–12 m in both groups), 90 (at 3–6 m in the AR group and at 6–12 m in both groups), and 120 min (at 0–3 m in the AR group and at 3–6 m and 6–12 m in both groups).

The DM remission was considered when the level of HbA1C was ≤ 6 gm% for at least 6 months. At 3 months 30.4% in AP and 41.5% in AR, at 6 months 42.9% in AP and 62.3% in AR, and at 12 months 60.7% in AP and 90.6% in AR of patients had DM remission. Logistic regression was used to study the association of DM remission with gastric emptying (at 0.5, 1, 1.5, and 2 h) at 12 months adjusted by BMI and %TWL at 12 months. The logistic regression model was statistically non-significant *χ*^2^(6) = 8.745, *p* value = 0.364. The model explained 18.4% (Nagelkerke *R*^2^) of the variance in DM remission and correctly classified 74.3% of cases. Increased gastric emptying (at 0.5, 1, 1.5, and 2 h) was not associated by increased incidence of DM remission (*p* value was 0.599, 0.053, 0.581, and 0.495 at 0.5, 1, 1.5, and 2 h respectively).

## Discussion

Laparoscopic sleeve gastrectomy (LSG) is regarded as one of the popular bariatric procedures nowadays. This is due to its feasibility, safety, and rapid learning curve in comparison to other procedures. LSG helps, in addition to weight loss, in remission of associated medical conditions such as diabetes mellitus (DM), hypertension, and dyslipidemia. However, there is still a controversy in standardization of the technique especially whether or not to resect or preserve the antrum. In addition, the effect of antrum resection on gastric emptying was not yet established as some studies report increase in gastric emptying [17], others report decreases in gastric emptying or no effect at all in comparison to LSG with antrum preservation. Also, there is not enough evidence on the effect of antrum preservation versus resection on the postoperative BMI and remission of DM in type II diabetic patients.

This study was designed to compare LSG with antrum preservation (AP) with LSG with antrum resection (AR) in diabetic patients with BMI 30–40 kg/m^2^. Patients with BMI 30–40 kg/m^2^ were selected as these patients were the best to benefit from LSG. The study included 56 patients in the AP group and 53 patients in the AR group.

Both groups showed significant decrease in weight and BMI and increase in %TWL at 3, 6, and 12 months postoperatively which was more significant in the AR group in comparison to the AP group. The difference between the two groups increased with time allowing AR to be significantly ahead of AP. Avlanmis et al. compared LSG with 2 cm and 5 cm from the pylorus using 36 F bougie and found that shorter distance from the pylorus was associated with better results in weight loss and maintenance of % of excess weight loss [18]. However, Garay et al. stated no difference in % of excess weight loss between antrum resection (2 cm from pylorus) and antrum preservation (5 cm from pylorus) using 33 F versus 42 F bougie [13]. Omarov et al. had a significant lower BMI in patients with antrectomy (2 cm from pylorus) in comparison with preserved antrum (6 cm from pylorus) but only at 3 months [19] while Obeidat et al. had a study with same lengths and report significant difference till 24 months [20]. Pereferrer et al. reported more weight loss with LSG 3 cm from pylorus in comparison with LSG 8 cm from pylorus [21]. Yormaz et al. showed better weight loss with antral resection (2 cm from pylorus) in comparison with preserved antrum (6 cm from pylorus) at 12 months with diminished differences at 24 months [22].

Both groups showed improvement of DM status as evidenced by the decrease in FBS, PPBS, HbA1C, and use oral hypoglycemics. FBS and PPBS improved significantly at 3, 6, and 12 months yet with no significant differences between the two groups. As regards the HbA1C, the AR group achieved significantly lower levels of HbA1C than the AP group although there was no significant difference in the use of oral hypoglycemics. Melissas and Daskalakis stated that altered gastric emptying and gut hormones after LSG share in the mechanism of type II DM remission [23]. DM remission may not be directly linked to antral resection, but reduction of BMI induced by LSG and alteration in gut hormones as rise in postprandial GLP-1 induce DM remission [24, 25] and as AR had better effect on BMI than AP; therefore, AR indirectly had more control of DM than AP. Vives et al. had better improvement in HbA1C levels at 12 months in diabetic patients with antral resection [15]. Some studies supported the metabolic theory which suggested that the increase of GLP-1 secretion induced by increase in the gastric emptying caused by LSG produces increase in insulin secretion that helps in DM remission [25]. If this theory was correct, there should be better DM remission in AP with rapid gastric emptying more than in AR with slower gastric emptying but yet the opposite was the case here. Besides, the logistic regression model failed to find a statistically significant increase in DM remission with increase in gastric emptying. Thus, this study suggested the weight loss theory which stated that the improvement of DM status was linked to decrease in BMI and not to the effect of increased gastric emptying and the increase in GLP-1 as the AR group with slower gastric emptying had more improvement of DM status than the AP group.

Both groups had accelerated gastric emptying at 30, 60, 90, and 120 min after consumption of Tc-99 m labeled standard semi-fluid meal postoperatively at 3, 6, and 12 months when compared with preoperative values. However, AP showed statistically significant accelerated rate of evacuation than AR at all time points as evidenced by T 1/2 which was the time needed by the stomach to empty half of the consumed standard meal. The pattern of gastric emptying in AP showed an accelerated mode through time which become faster by time with significant differences within group by repeated measure ANOVA in contrary to the AR that showed less tendency to be accelerated with non-significant differences within group by repeated measure ANOVA. Yet, the AR group had more incidence of GERD symptoms than AP although not statistically significant. In a study on LSG with antral preservation (6 cm from pylorus using 42 F bougie), Bernstine et al. found no difference before and after LSG in gastric emptying [26] in contrary to the results obtained by Johari et al. [27]. Melissas et al. reported accelerated gastric emptying after LSG with T 1/2 decreasing from 94.3 to 47.6 min in 11 patients after 6 months [6]. Baumann et al. stated that LSG 5–6 cm from the pylorus using 34 F bougie showed accelerated gastric emptying [28] and also in a study by Garay et al. [13]. Shah et al. reported increase transit time after LSG in type II diabetic patients [8]. Also, Nakane et al. concluded that the longer length the antrum is, the faster is the gastric emptying [29] which is similar to the results obtained in this study. Similar results were reported by Sioka et al. [30].

This study had large effect size (obtained by partial eta squared) and observed power of more than 80% in all recorded variables. The study was limited by the short-term results up to 1 year and by being powered to the primary outcome which was the gastric emptying.

Although prevalence of GERD symptoms was not statistically different, the AR group had threefold incidence at 6 and 12 months in comparison with the AP group. Since the study was not powered to show differences in GERD symptoms, this might be clinically significant.

## Conclusion

Laparoscopic sleeve gastrectomy (LSG) with antrum resection (2 cm from the pylorus) had significantly slower gastric emptying associated with less postoperative BMI, higher %TWL, and better control of type II DM in patients with BMI 30–40 kg/m^2^ in comparison with LSG with antral preservation (2 cm from the pylorus) with non-significant increase in incidence of GERD symptoms. Further studies to delineate the mechanisms involved with analysis of more variables are required to have full picture and standardize the LSG procedure.
